# Metagenomic analysis of mycobiome in wild and captivity Sumatran orangutans (*Pongo abelii*)

**DOI:** 10.5455/javar.2023.j673

**Published:** 2023-06-30

**Authors:** Safika Safika, Agustin Indrawati, Usamah Afif, Rahmat Hidayat, Titiek Sunartatie

**Affiliations:** Division of Medical Microbiology, School of Veterinary Medicine and Biomedical Sciences, IPB University, Bogor, Indonesia

**Keywords:** Captivity Sumatran orangutans, gut microbiota, mycobiome, *Pongo abelii*, wild Sumatran orangutans

## Abstract

**Objective:**

This study analyzes the mycobiome in wild and captive Sumatran orangutans.

**Materials and Methods:**

Nine orangutan feces samples from the wild and nine from captivity were divided into three repeats from 11- to 15-year-olds in good health. The Illumina platform for analysis of ITS bioinformatics was used according to the Qiime2 and CCMetagen approaches.

**Results:**

Wild Sumatran orangutans include 53% Ascomycota, 38% uncultured fungi, and 4% Basidiomycota. Orangutans in captivity are 57% Ascomycota, 26% uncultured fungi, and 2% Basidiomycota. Based on genus level, uncultured *Neurospora* (31%), *Penicillium *(10%), *Aspergillus* (3%), *Fusarium* (3%), *Candida* (2%), *Cutaneotrichosporon* (2%), and *Limonomyces* (2%) are found in wild orangutans. The most prevalent genus among captivity orangutans is *Aspergillus* (32%), followed by fungal sp. (11%), *Lasiodiplodia* (18%), *Devriesia* (2%), and *Sordariomycetes* (2%). According to the Chao1 diversity index and Shannon and Simpson, there was no significant difference between wild and captive Sumatran orangutans.

**Conclusion:**

*Neurospora* is unique to wild Sumatran orangutans, although *Aspergillus* predominates in captive orangutans. We hypothesize that the gut mycobiome of wild orangutans will resemble that of orangutans in captivity. The excellent range of food sources in the forest does not result in the prevalence of fungi in the typical gut microbiome.

## Introduction

Orangutans are one of Indonesia’s endemic primates. Orangutans are one of Southeast Asia’s few remaining giant apes (*Pongo *spp.). Orangutans are frugivores whose primary food source is fruit and insects [1,2]. Orangutan survival is highly dependent on forest conditions. Orangutans occupy two major Indonesian islands: Sumatra (*Pongo abelii* and *Pongo tapanuliensis*) and Kalimantan (*Pongo pygmaeus*) [3,4]. The Sumatran orangutan is an arboreal species that spends most of its time in trees. However, its lifestyle can be categorized as that of an explorer, settler, or nomad [5]. This is one of the distinctions between the daily habits of Kalimantan and Sumatran orangutans [6].

Sumatran orangutans are predominantly arboreal, or tree-dwelling or tree-working, mammals. The everyday activities of orangutans consist primarily of eating, relaxing, and social interaction. They are accustomed to spending most of their time eating in the morning and afternoon. The Sumatran orangutan’s eating habit involves tasting. Orangutans inhale the aroma and taste some of their food before ingesting it. If the orangutan does not like the food, it will discard it and search for other food. Orangutans engage in resting behaviors while sleeping at night and taking naps between activities. They typically construct their nests in the canopy of trees to avoid predators. Sumatran orangutans are semi-solitary animals. Typical orangutan social activity involves interactions between mother and offspring and young orangutans playing.

Humans and orangutans share similar genetic and physiological traits. Orangutan research is focused on protecting forests as orangutan habitats, while orangutan health has gotten little attention [7]. According to the study, these primates may be susceptible to bacterial, fungal, and parasitic infections that could spread widely among these species and to humans. Most orangutan research has centered on the creatures’ behavior, ecology, and physiology. The gut microbiome of primates is a complex collection of bacteria, archaea, viruses, protists, and fungi [8]. Even though the role of bacteria as causal contributors influencing host physiological development has been explored, the role of the mycobiome as causal contributors has yet to be investigated, and no studies on fungi have been published.

Studies have shown that fungi present in the gut microbiome of primates can control the immune responses of the host by either reducing or increasing local inflammation. Generally, fungi produce enzymes and antibiotics that aid the host’s metabolism. A healthy microbial environment in the digestive system stimulates the production of intestinal villi, which enhances the efficiency of the intestinal barrier and helps protect against harmful bacterial infections and nutrient absorption [9,10]. However, changes in gut microbial diversity are associated with gastrointestinal disorders and inflammation. Alterations in the metabolic activity of the gut can also lead to changes in the gut microbiota that are linked to poor health [11]. Therefore, it is crucial to identify the mycobiome of wild orangutans as a resource for research purposes to help conserve orangutans.

## Materials and Methods

### Ethical approval

This study obtained a permit and a written recommendation letter from Kementerian Lingkungan Hidup dan Kehutanan Direktorat Jenderal Konservasi Sumber Daya Alam dan Ekosistem. Nomor: SK 433/KSDAE/SET.3/KSA.2/8/2021.

### Sample collection

The samples were gathered from healthy 11 to 15-year-old orangutans (*P. abelii*) in the wild and in captivity. Randomly combining nine samples from orangutans in the wild and nine samples from orangutans in captivity yielded three replicates, each containing three samples. The orangutans to be sampled are in good health, and none of the orangutans in custody is undergoing therapy. The feces of newly defecated orangutans are collected in the middle to avoid environmental contamination, sealed in a plastic bag, and stored in a cold box until the laboratory reaches a temperature between 2°C and 10°C.

The feces of wild adult Sumatran orangutans from Gunung Leuser National Park in Southeast Aceh Regency were collected. Geographically, Gunung Leuser National Park spans latitude 903°02'50.5'' north and longitude 097°25'02.0'' east, encompassing a total area of 281,574.62 ha. Regarding terrain, conditions range from coastal regions (0 meters above sea level/masl) to mountainous regions (3,000 masl). Almost 80% of the terrain has a gradient greater than 40%. In this jungle, orangutans live naturally without human intervention in health management, diet, immunization, or worm treatment. Their diet comprises fruits gathered and ingested directly from trees, and they live from 15 to 40 m above the ground, where feces are identified. Orangutan feces were collected when the orangutans defecated early in the morning [4].

Meanwhile, fecal samples were taken from nine Sumatran orangutans in captivity at the Indonesian Safari Park in Bogor. This 168-hectare park is between 900 and 1,800 masl and has average temperatures between 16°C and 240°C. Orangutans in captivity were never administered antibiotics. The captive orangutans in this experiment had never received antibiotic therapy before.

### DNA extraction and analysis of 16S rRNA

The Qiagen DNeasy PowerSoil Kit was used to extract the feces of wild and captive Sumatran orangutans (Qiagen, Germantown, MD). Following the manual’s instructions, 250 mg of fecal samples were utilized to extract DNA. The internal transcribed spacer region (ITS) region was amplified by PCR using the primers ITS1F (5′-CTT GGT CAT TTA GAG GAA GTAA-3′) and ITS2 (5′-GCT GCG TTC ATC GAT GC-3′ with barcode) for 35 cycles at 95°C for 30 sec, 52°C for 30 sec, and 72°C for 30 sec [12]. All PCR studies utilized Phusion^®^ High-Fidelity PCR Master Mix (New England Biolabs).

### Preparation and sequencing

In the process from DNA samples to the final data, each step, including sample testing, PCR, purification, library creation, and sequencing, will affect the quality and amount of the data. In contrast, the data quality would immediately affect the findings of the information analysis. Quality control is performed at each stage of the process to ensure the precision and dependability of the sequencing data.

### ITS bioinformatics

DNA libraries generated with the NEBNext^®^ UltraTM DNA Library Prep Kit for Illumina and quantified using Qubit and quantitative Polymerase chain reaction) would be analyzed on the Illumina platform. The Illumina NovaSeq was utilized to sequence a 150-bp at a depth of 1,100. Based on the specific barcodes of each sample, paired-end reads were assigned to them, and the barcode and primer sequences were deleted. To merge paired-end readings, FLASH (V1.2.7) was utilized [13]. According to the Qiime2 (Version 2021.4) [14] quality-controlled procedure, the raw tags were subjected to quality filtering using specific filtering parameters to create high-quality clean tags [15]. Using the UCHIME algorithm, the tags were matched to the SILVA reference database (http://arb-silva.de/), and chimeric sequences were deleted [16]. The Effective Tags were finally secured.

Uparse software (Uparse v7.0.1090) evaluated sequences using all available tags [17]. The similarity between sequences categorized as the same Operational Taxonomic Unit (OTUs) was 97%. A representative sequence for each OTU was screened for further annotation. For each representative sequence, a query was conducted using Qiime2 (Version 2021.4) [14] in the Mothur method against the Small Subunit rRNA database of the SILVA Database (http://arb-silva.de/). For each taxonomic level, provide species annotation. (Threshold:0.81) [18]. The MUSCLE (Version 3.8.31) can swiftly compare numerous sequences to identify the phylogenetic connection of all OTU representative sequences [19]. Utilizing a standard sequence number related to the sample containing the minor sequences, the abundance information for OTUs was normalized. Based on these output normalized data, the following alpha diversity analysis was carried out, involving Chao1, Shannon, and Simpson, to examine the complexity of biodiversity for a sample utilizing alpha diversity. Each of these indexes was generated and shown in our samples using the R programming language (Version 2.15.3) [20].

Using clustering analysis and an evaluation of molecular variation to determine the commonalities between multiple specimens, a cluster tree was constructed. Principal Coordinates Analysis (PCoA) can be utilized to depict beta diversity [21]. The Bray-Curtis index and permutational multivariate analysis of variance (PERMANOVA) were utilized to determine whether or not the difference in microbial community structure between wild and captivity Sumatran orangutans is statistically significant [22].

We employed the CCMetagen approach to increase the accuracy and speed of metagenome classifiers. Pipeline greatly outperforms other commonly used tools in recognizing bacteria and fungi and can efficiently use the entire National Center for Biotechnology Information nucleotide collection as a reference for discovering species with insufficient genome data [23].

## Results

### Sequencing results

The composition of the gut microbiota was effectively studied by collecting feces samples from nine wild and captive Sumatran orangutans (*P. abelii*) aged 11-15 years and randomly combining them into three replicates of three samples each. When collecting samples of wild orangutans, we scoured the forest for trees that provided orangutans with food. After seeing the orangutans eating, we followed them until they defecated. The read counts data reveal a maximum of 328,145 reads (Exsitu3) and a minimum of 87,574 reads (Exsitu2), both of which are from captivity Sumatran orangutans, as shown in Table 1.

**Table 1.** Read counts from sample collections of wild and captivity Sumatran orangutans *(P. abelii).*

Sample ID	Sample type	Read counts
Wild1	Wild orangutan	259,398
Wild2	Wild orangutan	261,564
Wild3	Wild orangutan	265,495
Exsitu1	Captivity	275,280
Exsitu2	Captivity	87,574
Exsitu3	Captivity	328,145

### Fungi composition among Sumatran orangutans in the wild and captivity (P. abelii)

The relative frequency of different microbial taxa in wild Sumatran orangutans is 53% Ascomycota, 38% uncultured fungi, and 4% Basidiomycota. Sumatran orangutans’ captivity phyla are 57% Ascomycota, 26% uncultured fungi, and 2% Basidiomycota. Based on the results of OTU identification and taxonomic annotation According to CCMetagen, the total fungi at the genus level from the phylum Ascomycota and Basidiomycota in wild Sumatran orangutans are uncultured *Neurospora* (31%), *Penicillium *(10%), *Aspergillus* (3%), *Fusarium* (3%), *Candida* (2%), *Cutaneotrichosporon* (2%), and *Limonomyces* (2%). *Aspergillus* is the predominant genus among captivity orangutans (32%), followed by fungal species (11%), *Lasiodiplodia* (18%), *Devriesia* (2%), and *Sordariomycetes* (2%).

At the five species level of wild Sumatran orangutans (*P. abelii*), as determined by CCMetagen analysis, *Neurospora, Penicillium *sp*. SYFz-1, cf. Limonomyces roseipellis, uncultured Fusarium*, and *Candida albicans* were identified. In captivity, Sumatran orangutans, *Penicillium *sp*. SYFz-1, uncultured Fusarium, cf. Limonomyces roseipellis, C. albicans*, and *uncultured Aspergillus* were the most prevalent species (Tables 2 and 3).

### Alpha and beta variation among Sumatran orangutans (P. abelii)

To examine the microbial community makeup in each sample, sequences classified as the same OTUs shared 97% similarity on the Effective Tags of all samples. The rarefaction curve depicts the relationship between the number of species and the number of samples. Rarefaction can be used to verify if a sample has been sufficiently sequenced to represent its identity accurately. It can also be used to determine if a group of samples originates from the same community.

**Table 2.** Thirty prominent species from the phyla Ascomycota and Basidiomycota found in wild Sumatran orangutans (*P. abelii*) as determined by CCMetagen analysis.

No.	Phylum	Genus	Species	Score
1	Ascomycota	unk_g	*uncultured Neurospora*	1956,016
2	Ascomycota	*Penicillium*	*Penicillium *sp.* SYFz-1*	785,976
3	Basidiomycota	unk_g	*cf. Limonomyces roseipellis*	205,896
4	Ascomycota	unk_g	*uncultured Fusarium*	196,064
5	Ascomycota	*Candida*	*Candida albicans*	190,480
6	Basidiomycota	*Cutaneotrichosporon*	*Cutaneotrichosporon cutaneum*	111,526
7	Ascomycota	unk_g	*uncultured Golovinomyces*	71,486
8	Ascomycota	unk_g	*uncultured Ascomycota*	51,088
9	Ascomycota	*Trichoderma*	*Trichoderma longibrachiatum*	44,656
10	Ascomycota	unk_g	*uncultured Aspergillus*	30,666
11	Basidiomycota	*Cutaneotrichosporon*	*Cutaneotrichosporon jirovecii*	29,828
12	Ascomycota	*Neurospora*	*Neurospora calospora*	26,646
13	Basidiomycota	*Naganishia*	*Naganishia diffluens*	19,870
14	Ascomycota	*Pichia*	*Pichia kudriavzevii*	14,352
15	Ascomycota	*Kazachstania*	*Kazachstania pintolopesii*	12,704
16	Ascomycota	*Phoma*	*Phoma herbarum*	7,172
17	Ascomycota	*Cladosporium*	*Cladosporium *sp*. PS4B-F9*	6,098
18	Ascomycota	*Microsphaeropsis*	*Microsphaeropsis proteae*	5,756
19	Ascomycota	*Candida*	*Candida parapsilosis*	4,450
20	Ascomycota	*Aplosporella*	*Aplosporella javeedii*	4,426
21	Ascomycota	*Aspergillus*	*Aspergillus flavus*	4,374
22	Basidiomycota	*Naganishia*	*Naganishia liquefaciens*	3,952
23	Ascomycota	*Neomicrosphaeropsis*	*Neomicrosphaeropsis minima*	3,908
24	Ascomycota	*Aspergillus*	*Aspergillus oryzae*	2,856
25	Basidiomycota	*Rhodotorula*	*Rhodotorula kratochvilovae*	2,388
26	Ascomycota	*Talaromyces*	*Talaromyces funiculosus*	2,044
27	Ascomycota	*Cladosporium*	*Cladosporium *sp*.*	1,584
28	Ascomycota	*Briansuttonomyces*	*Briansuttonomyces eucalypti*	1,500
29	Mucoromycota	*Rhizopus*	*Rhizopus oryzae*	1,040
30	Ascomycota	*Saccharomyces*	*Saccharomyces cerevisiae*	1,028

According to the Chao1 diversity index, Shannon and Simpson found no significant difference between wild and captive Sumatran orangutans regarding microbial diversity. Chao1 has a *p*-value of 0.635 and a *T*-test statistic of 0.585; Shannon has a *p*-value of 0.798 and a *T*-test statistic of −0.386; and Simpson has a *p*-value of 0.891 and a *T*-test statistic of 0.032.

Beta diversity is a comprehensive comparison of microbiomes’ diversity based on their diversity. Therefore, beta diversity measurements are used to evaluate the differences between microbiomes. A cluster tree was constructed using clustering analysis to examine the commonalities among multiple specimens. PCoA can be utilized to depict beta diversity. Optimizing the linear correlation between sample values is the objective of the PCoA. The distance technique employs the Bray-Curtis index and PERMANOVA to establish statistical significance in this study. According to the PERMANOVA results, the R-squared value is 0.14629, and the *p*-value is 0.90. The microbial community composition of Sumatran orangutans in the wild and in captivity does not differ significantly. The cluster analysis aims to generate a tree diagram where the most comparable data points are placed on nearby branches. The investigation findings indicate that the hierarchical clusters of Exsitu1 are more comparable to Exsitu3 than Exsitu2.

**Table 3.** Thirty prominent Ascomycota and Basidiomycota species isolated from captive Sumatran orangutans (*P. abelii*) using CCMetagen analysis.

No.	Phylum	Genus	Species	Score
1	Ascomycota	*Penicillium*	*Penicillium *sp*. SYFz-1*	978,186
2	Ascomycota	unk_g	*uncultured Fusarium*	299,060
3	Basidiomycota	unk_g	*cf. Limonomyces roseipellis*	264,696
4	Ascomycota	*Candida*	*Candida albicans*	241,814
5	Basidiomycota	*Aspergillus*	*uncultured Aspergillu*s	118,244
6	Ascomycota	unk_g	*uncultured Golovinomyces*	71,868
7	Ascomycota	*Candida*	*Candida albicans*	66,458
8	Ascomycota	*Trichoderma*	*Trichoderma longibrachiatum*	64,738
9	Ascomycota	*Cutaneotrichosporon*	*Cutaneotrichosporon cutaneum*	39,958
10	Basidiomycota	*Cutaneotrichosporon*	*Cutaneotrichosporon jirovecii*	34,206
11	Basidiomycota	*Naganishia*	*Naganishia diffluens*	23,908
12	Ascomycota	*Kazachstania*	*Kazachstania pintolopesii*	13,944
13	Ascomycota	*Cladosporium*	*Cladosporium *sp*. PS4B-F9*	13,524
14	Ascomycota	*Pichia*	*Pichia kudriavzevii*	12,438
15	Ascomycota	*Phoma*	*Phoma herbarum*	10,606
16	Ascomycota	*Microsphaeropsis*	*Microsphaeropsis proteae*	6,728
17	Ascomycota	unk_g	*uncultured Neurospora*	5,882
18	Ascomycota	*Candida*	*Candida parapsilosis*	5,428
19	Ascomycota	*Aspergillus*	*Aspergillus flavus*	5,264
20	Ascomycota	*Neomicrosphaeropsis*	*Neomicrosphaeropsis minima*	5,172
21	Ascomycota	*Fusarium*	*Fusarium oxysporum*	4,644
22	Ascomycota	*Aplosporella*	*Aplosporella javeedii*	4,538
23	Ascomycota	*Aspergillus*	*Aspergillus oryzae*	3,584
24	Basidiomycota	*Rhodotorula*	*Rhodotorula kratochvilovae*	2,970
25	Ascomycota	*Talaromyces*	*Talaromyces funiculosus*	2,698
26	Basidiomycota	*Malassezia*	*Malassezia furfur*	2,618
27	Basidiomycota	*Naganishia*	*Naganishia liquefaciens*	2,614
28	Ascomycota	*Microsphaeropsis*	*Microsphaeropsis olivacea*	1,948
29	Ascomycota	*Fusariella*	*Fusariella *sp*. MFLUCC 15-0844*	1,946
30	Ascomycota	*Briansuttonomyces*	*Briansuttonomyces eucalypti*	1,500

## Discussion

This study is the first to investigate the mycobiome of both wild and captive Sumatran orangutans, although the fungal aspect of gut microbiota has received less attention compared to bacteria. Despite individual variations in gut microbiota, such as age, sex, geography, diet, and health, fungal commensals in the gut play critical roles in the mammalian immune response. This research sheds light on the dietary and digestive adaptations of orangutans, as the diversity of their gut mycobiome is comparable to that of other primates. Interestingly, the diversity of the gut mycobiome of wild orangutans is similar to that of captive orangutans, despite the wider range of food sources available in the wild.

The gut mycobiome plays a vital role in digesting challenging-to-digest substances such as fiber and oligosaccharides. Through fermentative metabolism, the mycobiome generates various metabolites like short- and medium-chain fatty acids and gases. It also plays a significant role in safeguarding the epithelial barrier against pathogens and boosting immunity by activating T-cell 17 immune mechanisms. Thus, maintaining mucosal homeostasis is one of the critical functions of the gut mycobiome [24,25].

The ITS region of fungal rRNA varies in length among different fungal species, which can introduce bias when using next-generation sequencing technology. Moreover, ITS variation may not always be sufficient to distinguish between species, and few references are available to match sequences to organisms [26]. Although ITS1 was previously considered the best target for fungal profiling, recent research has shown that ITS2 is more accurate. However, ITS1 and ITS2 produce identical community structure patterns regarding clustering and taxonomic capabilities [27]. Based on the OTU findings, there are no species-level differences between captive and wild Sumatran orangutans’ gut mycobiomes. Identified fungi in both groups include *Penicillium* sp*. SYFz-1, cf. Limonomyces roseipellis, uncultured Fusarium,* and *C. albicans*. However, wild orangutans had uncultured *Neurospora*, while captive Sumatran orangutans had the highest levels of uncultured *Aspergillus*.

Sumatran orangutans consume various food items, including fruits, young leaves, flowers, honey, shoots, stems, seeds, nuts, bamboo, mushrooms, bark, termites, and ant eggs. These food items are rich in cellulose and hemicellulose, which primates cannot digest independently due to a lack of the cellulase enzyme. Therefore, they rely on their gut microbiome to produce this enzyme. Fungi found in the guts of wild and captive Sumatran orangutans are believed to aid in metabolic processes, particularly the digestion of cellulose and hemicellulose. As a result, the mycobiomes of wild and captive Sumatran orangutans were not significantly different. Similar findings have been reported in studies of Tibetan macaques and Lemurs, which also had *Aspergillus, Penicillium*, and *Fusarium* fungi in their guts. These fungus genera produce enzymes that are useful for breaking down cellulose biomass in plant diets rich in cellulose and hemicellulose [28,29]. Furthermore, *Neurospora crassa*, a species in the genus *Neurospora*, is known for possessing regulatory genes that express cellulase genes, cellulase activity, and cellulase secretion [30].

*Neurospora* is a type of filamentous fungus belonging to the Ascomycota phylum. The name Neurospora refers to the appearance of its spores, which have striations resembling axons. This fungus is widely used in cellular research to study aspects of eukaryotic biology, such as development and differentiation [31]. In addition, *Neurospora* is a valuable model organism for studying epigenetic phenomena, as it has various genome defense and epigenetic mechanisms. *Neurospora* is the only organism with repeat-induced point mutations, a unique feature among fungi [32]. While there is no direct evidence that *Neurospora* has a secondary metabolism, some gene sequences have been identified as potential candidates for secondary metabolite production [33].

*Candida albicans* has been found in both wild and captive Sumatran orangutans. While most research on the mycobiome has focused on opportunistic pathogens like *C. albicans*, it is actually a common fungus in the gut of healthy humans and animals, making it a typical member of the human gut microbiota [34–38]. Since *C. albicans* lacks a significant reservoir in the environment, it is believed to have coevolved extensively with its host and other microbes. Although *Candida* can also colonize other body parts, such as the mouth, skin, and vagina, it is a frequent cause of mucosal disease in otherwise healthy individuals [39,40]. However, it can also enter the bloodstream from the gut and cause invasive [41,42] and life-threatening infections in immunocompromised individuals such as organ transplant recipients or cancer patients undergoing chemotherapy [36].

The mycobiome in the gastrointestinal tract, which is estimated to be around 0.1% of the total gut microbiome in humans [43,44], likely has a similar proportion in orangutans. The mycobiome is essential for maintaining intestinal health, immune system development, and disease pathogenesis [25,44]. An imbalance in the composition of the gut microbial community, including the mycobiome, known as dysbiosis, has been linked to various diseases, such as autoimmune disorders, metabolic disorders, neurological disorders, and cancer. The colonization and growth of opportunistic fungal pathogens in the gut can cause dysregulated immune responses and affect disease progression. *Candida *spp*.* has been found to influence the gut bacterial microbiome’s assembly and function through various mechanisms such as cellular contact, competition, collaboration for nutrients, and the production of secondary metabolites and antimicrobial peptides. These interactions between gut fungi, bacteria, and host immunity are essential for maintaining human immune homeostasis, which can influence overall health and disease outcomes [45–47].

The variability of the gut mycobiome is influenced by dietary and environmental factors and host factors such as genetics, age, sex, use of antibiotics, and antifungals. The gut mycobiome shows higher variability between individuals and instability over time than the gut bacterial microbiome. Individuals with different nutritional patterns have different gut mycobiome compositions. A carbohydrate-rich diet is associated with an increased abundance of *Candida* spp. In contrast, a protein-enriched diet is associated with a decreased abundance of *Candida *spp. and *Methanobrevibacter* bacteria in healthy individuals [44,48,49]. Overall, the gut mycobiome is a complex and dynamic ecosystem that is influenced by a variety of factors, and more research is needed to fully understand its role in human and animal health.

However, the study has limited samples of wild orangutans that reside in tall trees and has detected a population of approximately 30 wild orangutans dispersed across hundreds of hectares of forest. It is important to continue research on the mycobiome of wild orangutans and expand the sample size to gain a more comprehensive understanding of the gut microbiome in these animals. Such studies could also provide valuable insights into the role of the gut microbiome in the evolution of orangutans and their adaptation to changing environments. This information could also be used to develop strategies to support the conservation of orangutan populations, as changes in diet and habitat could affect the composition and function of their gut microbiomes, including the mycobiome.

## Conclusion

Wild Sumatran orangutans are composed of 53% Ascomycota, 38% uncultured fungi, and 4% Basidiomycota. In captivity, 57% of the orangutan phyla are Ascomycota, 26% are uncultured fungi, and 2% are Basidiomycota. *Neurospora* is unique to wild Sumatran orangutans, although *Aspergillus* predominates in captive orangutans. We predict that the gut mycobiome of wild orangutans will be similar to that of captive orangutans. The greater diversity of food sources in the forest does not result in the presence of fungi in the normal microbiome of the gut flora. The synergistic, symbiotic, or antagonistic interactions between fungal and bacterial microbiota members should be understood.

## List of abbreviations

GI, gastrointestinal; PERMANOVA, permutational multivariate analysis of variance; ITS, internal transcribed spacer region; masl, meters above sea level; OTU, Operational Taxonomic Unit; PCoA, Principal Coordinates Analysis
